# Decreased PPARgamma in the trigeminal spinal subnucleus caudalis due to neonatal injury contributes to incision-induced mechanical allodynia in female rats

**DOI:** 10.1038/s41598-022-23832-3

**Published:** 2022-11-11

**Authors:** Jo Otsuji, Yoshinori Hayashi, Suzuro Hitomi, Chihiro Soma, Kumi Soma, Ikuko Shibuta, Koichi Iwata, Tetsuo Shirakawa, Masamichi Shinoda

**Affiliations:** 1 Department of Pediatric Dentistry, Nihon University School of Dentistry, 1-8-13 Kandasurugadai, Chiyoda-ku, Tokyo, 101-8310 Japan; 2 Department of Physiology, Nihon University School of Dentistry, 1-8-13 Kandasurugadai, Chiyoda-ku, Tokyo, 101-8310 Japan

**Keywords:** Chronic pain, Chronic pain

## Abstract

Whisker pad skin incision in infancy causes the prolongation of mechanical allodynia after re-incision in adulthood. A recent study also proposed the importance of sex differences in pain signaling in the spinal cord. However, the sex difference in re-incision-induced mechanical allodynia in the orofacial region is not fully understood. In the rats that experienced neonatal injury in the whisker pad skin, the mechanical allodynia in the whisker pad was significantly prolonged after re-incision in adulthood compared to sham injury in infancy. No significant sex differences were observed in the duration of mechanical allodynia. The duration of mechanical allodynia in male rats was shortened by intracisternal administration of minocycline. However, minocycline had no effects on the duration of mechanical allodynia in female rats. In contrast, intracisternal administration of pioglitazone markedly suppressed mechanical allodynia in female rats after re-incision. Following re-incision, the number of peroxisome proliferator-activated receptor gamma (PPARgamma)-positive cells were reduced in the trigeminal spinal subnucleus caudalis (Vc) in female rats that experienced neonatal injury. Immunohistochemical analyses revealed that PPARgamma was predominantly expressed in Vc neurons. Pioglitazone increased the number of PPARgamma-positive Vc neurons in female rats whose whisker pad skin was incised in both infancy and adulthood stages. Pioglitazone also upregulated heme oxygenase 1 and downregulated NR1 subunit in the Vc in female rats after re-incision. Together, PPARgamma signaling in Vc neurons is a female-specific pathway for whisker pad skin incision-induced mechanical allodynia.

## Introduction

Tissue injury that is experienced in the fetal period and childhood due to surgery or an accident alters the somatosensory system. Accumulating animal studies demonstrated that alteration of the neuronal network in the central nervous system (CNS) caused by neonatal injury maintains till adulthood. The activity of gamma-aminobutyric acid (GABA)-ergic neurons in the spinal dorsal horn was dramatically reduced in adulthood when an animal suffered an injury in the hind paw on postnatal day 3^1^. Furthermore, nociceptive stimulation of the hind paw 3 days after birth changes the gene profile in the spinal dorsal horn, which was maintained until adulthood^2^. Functional alterations in glial cells such as microglia in adulthood were also caused by tissue injury in infancy^3,4^. After re-incision in the hind paw of the adult rats that experienced neonatal injury in the hind paw, microglia release brain-derived neurotrophic factor (BDNF) which elicits long-term potentiation of C-fiber-mediated synaptic responses in the spinal dorsal horn, culminating in the maintenance of excessive neuronal activity^5^. BDNF also causes the transformation of inhibitory GABAergic neurotransmission into excitatory one by the reduction of K^+^/Cl^-^ cotransporter in lamina I spinal neurons^6^. Accordingly, neonatal tissue injury primes the alteration of a spinal neuronal circuit in adulthood.

It is reported that the incidence rate of severe pain such as neuropathic pain in women is higher than in men, and these sex differences in pain signaling have been long proposed in the clinical setting^7^. Using an animal model of neuropathic pain, the CNS mechanisms involved in sex differences in pain signaling have been clarified by focusing on immune cells. The occurrence of neuropathic pain in male mice is dependent on multiple inflammatory factors from microglia in the spinal dorsal horn^8^. By contrast, that in female mice depends on the infiltration of activated T cells into the spinal dorsal horn^8^. In this research, pioglitazone, an agonist of peroxisome proliferator-activated receptor-gamma (PPARγ), ameliorates mechanical allodynia in female mice but not in male mice by the inhibition of T cell activation in the spinal dorsal horn. Even in the contribution of T cells in severe pain, the existence of T cells in the spinal dorsal horn is still a matter of controversy. Following sciatic nerve injury, the number of CD3- or CD4-positive T cells was increased in the spinal dorsal horn^9,10^. Besides, mechanical allodynia was not elicited in *Rag1*^*-/-*^ mice and *Rag2*^*-/-*^ mice which lack T cells and B cells^10,11^. In contrast, there are a few green fluorescence protein (GFP) and CD3 double-positive cells in the spinal cord after L4 spinal nerve transection in bone marrow chimeric mice that were transplanted GFP-positive bone marrow^12^. According to the above-mentioned facts, there may be a spinal cord infiltrating T cell-independent pathway in the development of mechanical allodynia in female animals. However, the details are still unclear.

PPARγ is a transcription factor that is classified as a nuclear receptor superfamily and formed heterodimer with retinoid X receptor^13^. PPARγ regulates the transcription of several genes and is primarily involved in the differentiation of adipocytes^13^. As mentioned above, PPARγ regulates the function of T cells, and its agonist ameliorates neuropathic pain in female mice^8^. In addition, PPARγ agonist ameliorates chronic pain caused by spinal cord injury in only female rats^14^. However, the detailed mechanisms of analgesic effects of PPARγ agonists are not clarified.

We previously reported that an increased number of Vc microglia is required for the prolonged mechanical allodynia caused by whisker pad skin re-incision in male rats that experienced neonatal incision in the whisker pad skin^4^. Given the involvement of microglia in pain signaling in the orofacial region in male rats, sex differences in pain signaling may be observed in the prolongation of skin incision-induced mechanical allodynia in rats with neonatal incision. The purpose of this study was to examine if there is a sex difference in the prolongation of mechanical allodynia by whisker pad skin re-incision in adult rats with neonatal incision and if PPARγ is involved in this mechanism.

## Results

### Effect of sex difference on incision-induced mechanical allodynia

**Figure 1:**
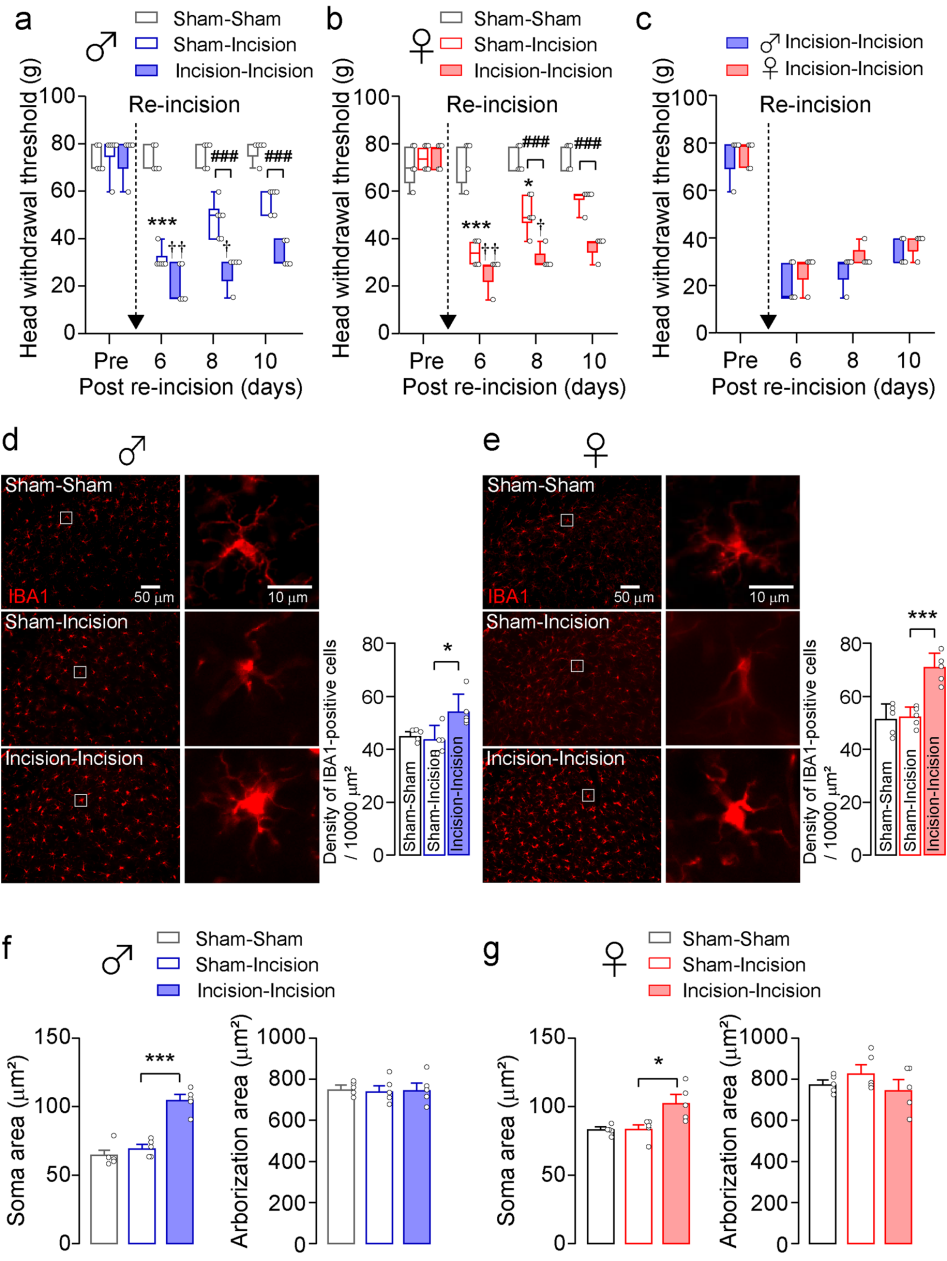
Microglia are activated in the trigeminal spinal subnucleus caudalis (Vc) in both male and female rats after whisker pad skin re-incision. (**a, b, c**) Time course of head withdrawal threshold (HWT) of male rats (**a**), female rats (**b**), and both male and female rats (**c**) after whisker pad skin re-incision. Broken arrows indicate that the whisker pad underwent skin re-incision at 7 weeks of age. “Pre” indicate the day before skin incision. Sham-Sham: rats with sham operation at both infancy and adulthood; Sham-Incision: rats which suffered sham operation in infancy were incised in adulthood; Incision-Incision: rats which suffered an incision in the whisker pad skin were incised in adulthood. Boxes show the 25th–75th percentiles with the median value indicated as a line within each box. Whiskers indicate the 10th and 90th percentiles of the data. n = 5 rats in each, Friedman's test post hoc Dun’s test (***p = 0.0003, vs. Sham-Incision Pre; †*p* = 0.0303, ††*p* = 0.00208, vs. Incision-Incision Pre); Generalized estimating equations (GEE) method post hoc Bonferroni’s test (###*p* < 0.0001, Sham-Incision vs. Incision-Incision) in (**a**), Friedman's test post hoc Dun’s test (*p = 0.0417, ***p = 0.0002, vs. Sham-Incision Pre; †*p* = 0.0417, ††*p* = 0.002, vs. Incision-Incision Pre); GEE post hoc Bonferroni’s test (###*p* < 0.0001, Sham-Incision vs. Incision-Incision) in (**b**). (**d, e)** The representative images of IBA1 immunofluorescence in the Vc. The right images are enlarged images of insets in the left images. Scale bars = 50 µm (left images) and 10 µm (right images). The columns represent the mean number of IBA1-positive cells in the Vc 10 days after skin re-incision. Data represent the mean ± SEM. n = 5 in each, one-way ANOVA post hoc Tukey’s test, *p = 0.0118 in (**d**), ***p = 0.0003 in (**e**). (**f, g**) Soma area and arborization area of Vc microglia in male (**f**) and female rats (**g**). The columns represent the mean soma area and arborization area of microglia in the Vc 10 days after skin re-incision. Data represent the mean ± SEM. n = 5 in each, one-way ANOVA post hoc Tukey’s test, ***p < 0.001 in (**f**), ***p = 0.0123 in (**g**).

To evaluate sex differences in pain signaling, we made an incision in the whisker pad skin in both male and female pups and re-injured it at 7 weeks after birth, and mechanical stimulation was applied to the whisker pad skin. In both sexes, no significant change in head withdrawal threshold (HWT) was observed in sham-injured rats with sham incision in infancy (Sham-Sham group) throughout the experimental period (male: Friedman’s test post hoc Dunn’s test, *p* = 0.5000, Fig. 1a; female: Friedman’s test post hoc Dunn’s test, *p* = 0.8750, Fig. 1b). Skin incision in adulthood caused a significant reduction in HWT on day 6 after re-incision in rats who suffered sham incision in infancy (Sham-Incision group) in both sexes (male: Friedman’s post hoc Dunn’s test, *p* < 0.001, Fig. 1a; female: Friedman’s post hoc Dunn’s test, *p* < 0.001; Fig. 1b). Skin incision in adulthood extended the duration of HWT decline after re-incision in rats with neonatal incision (Incision-Incision group) in both sexes compared to the Sham-Incision group (male: generalized estimated equation (GEE) post hoc Bonferroni’s test, *p* = 0.003, df = 1, Wald chi-square = 8.641, Fig. 1a; female: GEE post hoc Bonferroni’s test, *p* = 0.002, df = 1, Wald chi-square = 9.854, Fig. 1b). The duration of HWT decline after re-incision was unchanged between male and female rats with neonatal incision (GEE post hoc Bonferroni’s test, *p* = 0.086, df = 1, Wald chi-square = 2.945; Fig. 1c).

### Microglia do not contribute to the prolongation of HWT decline in female rats

**Figure 2:**
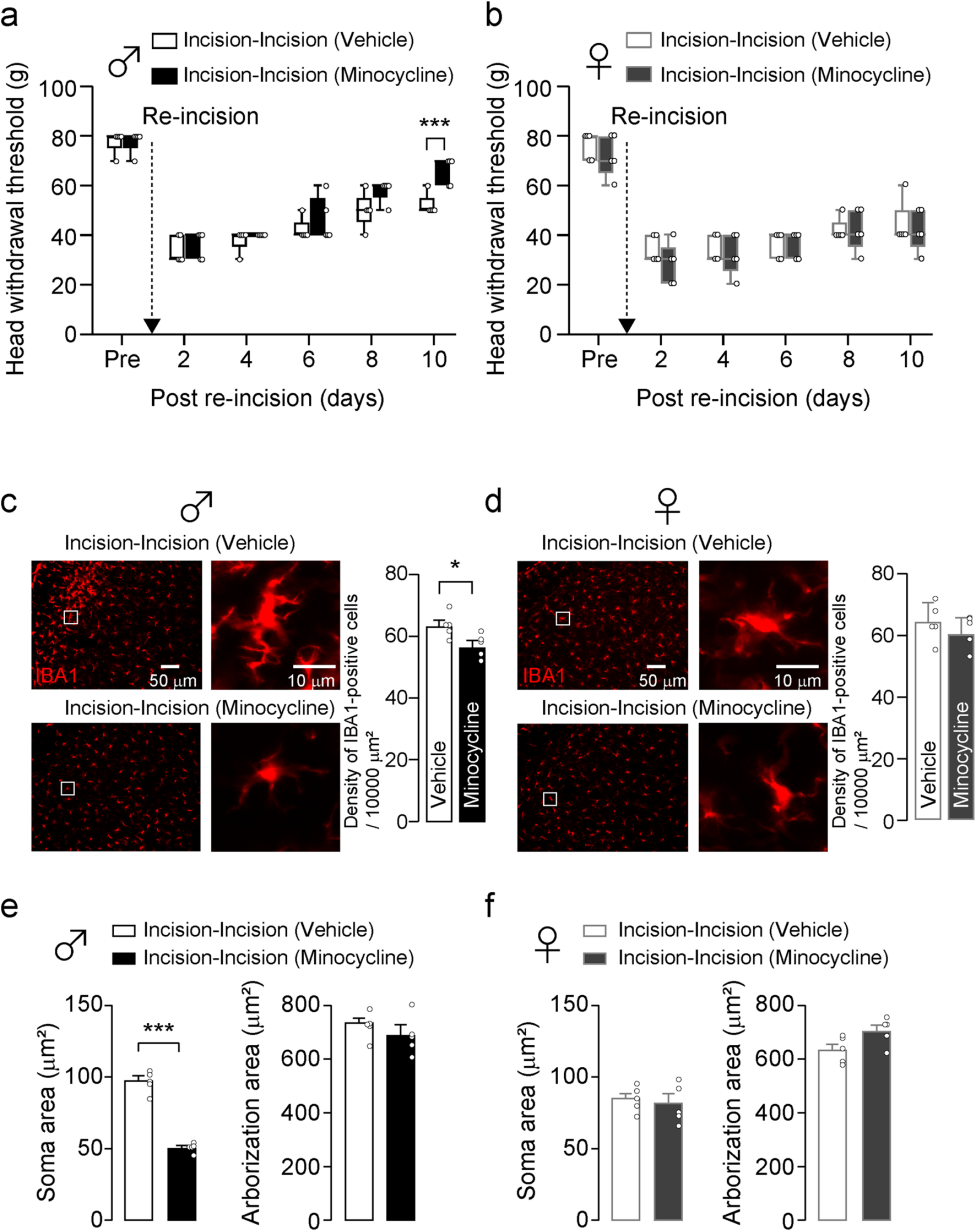
The effects of minocycline on incision-induced prolonged HWT decline and the number of Vc microglia of both male and female rats. (**a, b**) Time course of HWT of male rats (**a**) and female rats (**b**) after whisker pad skin re-incision. Vehicle or minocycline were continuously delivered in the cisterna magna by an osmotic pump before whisker pad skin re-incision. Broken arrows indicate that the whisker pad underwent skin re-incision at 7 weeks of age. “Pre” indicate the day before skin incision. Boxes show the 25th–75th percentiles with the median value indicated as a line within each box. Whiskers indicate the 10th and 90th percentiles of the data. n = 5 rats in each, GEE method post hoc Bonferroni’s test (***p < 0.001, Vehicle vs. Minocycline) (**c, d**) The representative images of IBA1 immunofluorescence in the Vc. The right images are enlarged images of insets in the left images. Scale bars = 50 µm (left images) and 10 µm (right images). The columns represent the mean number of IBA1-positive cells in the Vc 10 days after whisker pad skin re-incision. Data represent the mean ± SEM. n = 5 in each, unpaired t-test, *p = 0.0269 in (**c**). (**e, f**) Soma area and arborization area of Vc microglia in male (**e**) and female rats (**f**). The columns represent the mean soma area and arborization area of microglia in the Vc 10 days after skin re-incision. Data represent the mean ± SEM. n = 5 in each, unpaired t-test, **p < 0.001 in (**e**).

It is known that microglia are one of the important factors in sex differences in pain signaling^8,15,16^. We, therefore, assessed the contribution of microglia in the trigeminal spinal subnucleus caudalis (Vc) in the prolongation of HWT decline using immunohistochemical analysis. The density of ionized calcium-binding adapter molecule 1 (IBA1)-positive cells in the Vc was unchanged between the Sham-Sham group and the Sham-Incision group in both sexes (male: one-way ANOVA post hoc Tukey’s test, *p* = 0.5472, Fig. 1d; female: one-way ANOVA post hoc Tukey’s test, *p* = 0.9533, Fig. 1e). Following skin re-incision, the density of IBA1-positive cells in the Vc in the Incision-Incision group was significantly increased compared to the Sham-Incision group in both sexes (male: one-way ANOVA post hoc Tukey’s test, *p* = 0.0118, Fig. 1d; female: one-way ANOVA post hoc Tukey’s test, *p* = 0.0003; Fig. 1e). The soma area of Vc microglia in the Incision-Incision group was significantly increased compared to the Sham-Incision group in both sexes (male: one-way ANOVA post hoc Tukey’s test, *p* < 0.001, Sham-Incision vs. Incision-Incision; female: one-way ANOVA post hoc Tukey’s test, *p* = 0.0123, Sham-Incision vs. Incision-Incision; Fig. 1f,g). No significant sex differences in the arborization area were observed (Fig. 1f,g). These results imply that microglia were functionally changed in the Vc after skin re-incision in the Incision-Incision group of both sexes. To further evaluate the contribution of Vc microglia in the prolonged duration of HWT decline after skin re-incision, we continuously delivered minocycline, an inhibitor of microglial activation, into the cisterna magna before skin re-incision. Minocycline administration significantly increased the HWT in male rats 10 days after skin re-incision compared to vehicle-treated rats (GEE post hoc Bonferroni’s test, *p* = 0.007, df = 1, Wald chi-square = 7.401; Fig. 2a). On the other hand, minocycline did not affect the HWT in female rats after skin re-incision (GEE post hoc Bonferroni’s test, *p* = 0.525, df = 1, Wald chi-square = 0.403; Fig. 2b). The density of IBA1-positive cells in the Vc of the Incision-Incision group was significantly reduced by minocycline administration in male rats (unpaired t-test, *p* = 0.0269, t_(8)_ = 2.705; Fig. 2c). On the other hand, the density of IBA1-positive cells in the Vc of female rats was unaltered by minocycline administration (unpaired t-test, *p* = 0.3887, t_(8)_ = 0.9114; Fig. 2d). Minocycline treatment decreased the soma area of Vc microglia in Incision-Incision male rats but not in female rats (male: unpaired t-test, *p* < 0.001, t_(8)_ = 12.82; female: unpaired t-test, *p* = 0.6257, t_(8)_ = 0.5072; Fig. 2e,f). In contrast, the arborization area of Vc microglia was unaltered by minocycline administration in both male and female rats (male: unpaired t-test, *p* = 0.3743, t_(8)_ = 0.9409; female: unpaired t-test, *p* = 0.0577, t_(8)_ = 2.214; Fig. 2e,f).

### Effect of pioglitazone on incision-induced mechanical allodynia

**Figure 3:**
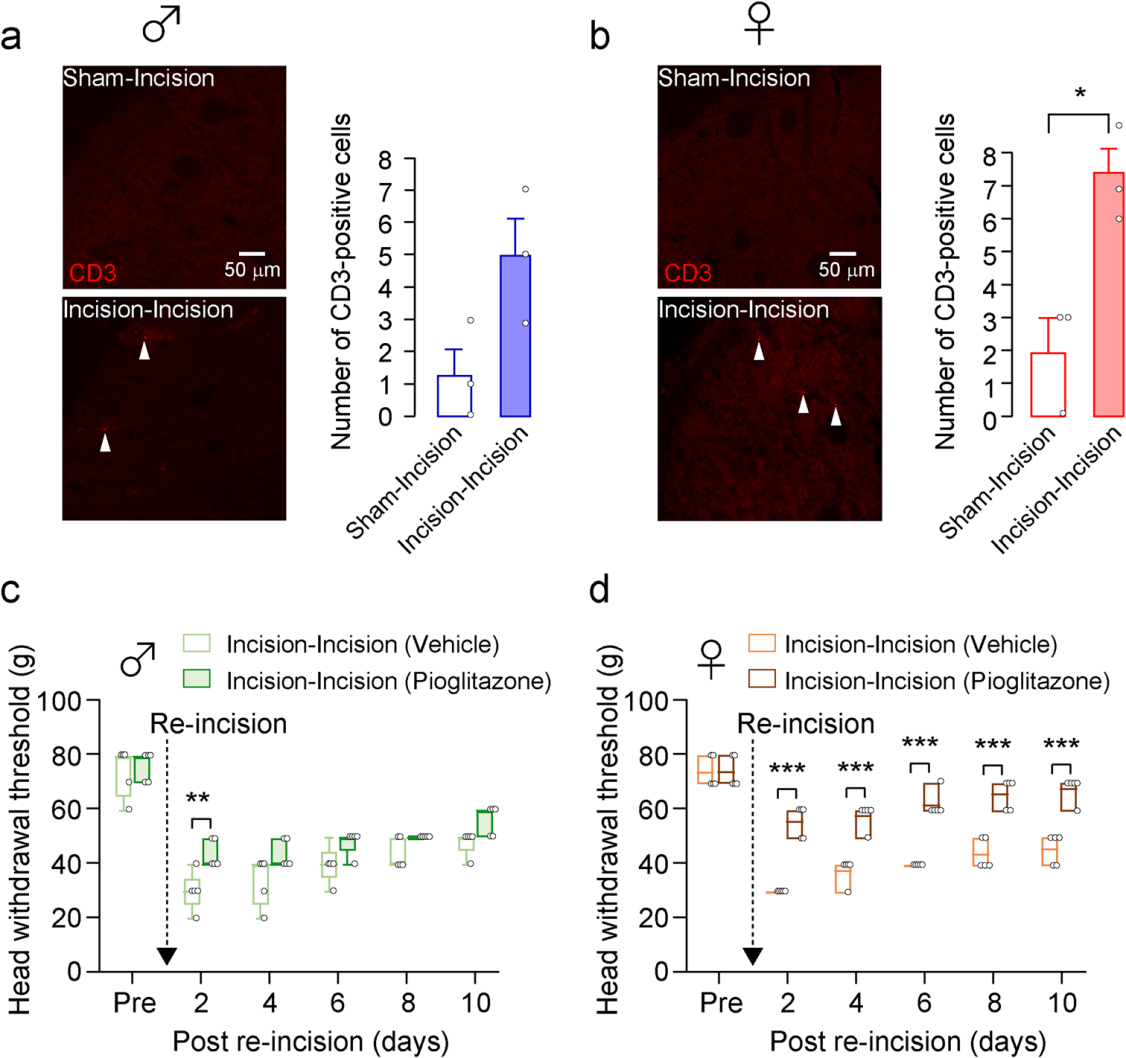
Infiltration of T cells in the Vc and the effects of pioglitazone on HWT after whisker pad skin re-incision in male and female rats. (**a, b**) The images showing CD3-positive cells in the Vc of male (**a**) and female rats (**b**) 10 days after whisker pad skin re-incision. Scale bars = 50 µm. The columns represent the mean number of CD3-positive cells in the Vc 10 days after whisker pad skin re-incision. Arrowheads indicate CD3-positive cells. Data represent the mean ± SEM. n = 5 in each, unpaired t-test, *p = 0.0161 in (**b**). (**c, d**) Time course of HWT of male rats (**c**) and female rats (**d**) after whisker pad skin re-incision. Vehicle and pioglitazone were continuously delivered in the cisterna magna by an osmotic pump before whisker pad skin re-incision. Broken arrows indicate that the whisker pad underwent skin re-incision at 7 weeks of age. “Pre” indicate the day before skin incision. Boxes show the 25–75th percentiles with the median value indicated as a line within each box. Whiskers indicate the 10th and 90th percentiles of the data. n = 5 rats in each, GEE method post hoc Bonferroni’s test (**p = 0.004 in (**c**), ***p < 0.001 in (**d**), Vehicle vs. Pioglitazone).

Infiltrated T cells in the spinal cord are required for the induction of mechanical allodynia after spared nerve injury in female rats^8^. We, therefore, investigated the T cell infiltration in the Vc after skin re-incision. We observed a few CD3-positive cells in the Vc of the sham-incision group of both sexes (Fig. 3a). Following skin re-incision, the number of CD3-positive cells was significantly increased in female rats but not in male rats (unpaired t-test, *p* = 0.0651, t_(4)_ = 2.524, Fig. 3a; unpaired t-test, *p* = 0.0184, t_(7)_ = 3.057, Fig. 3b). However, the increase in the number of CD3-positive cells was very slight. We next analyzed the effect of pioglitazone on incision-induced mechanical allodynia in male and female rats. Pioglitazone was continuously delivered in the cisterna magna of the Incision-Incision group before skin re-incision. In male rats, pioglitazone suppressed the decrease in HWT only on day 2 after skin re-incision (GEE post hoc Bonferroni’s test, *p* < 0.001, df = 1, Wald chi-square = 13.921; Fig. 3c). On the other hand, pioglitazone significantly suppressed the HWT decline after skin re-incision in female rats throughout the experimental period (GEE post hoc Bonferroni’s test, *p* < 0.001, df = 1, Wald chi-square = 137.544; Fig. 3d).

### Contribution of peroxisome proliferator-activated receptor gamma (PPARγ)-positive neurons in the Vc to incision-induced mechanical allodynia.

**Figure 4:**
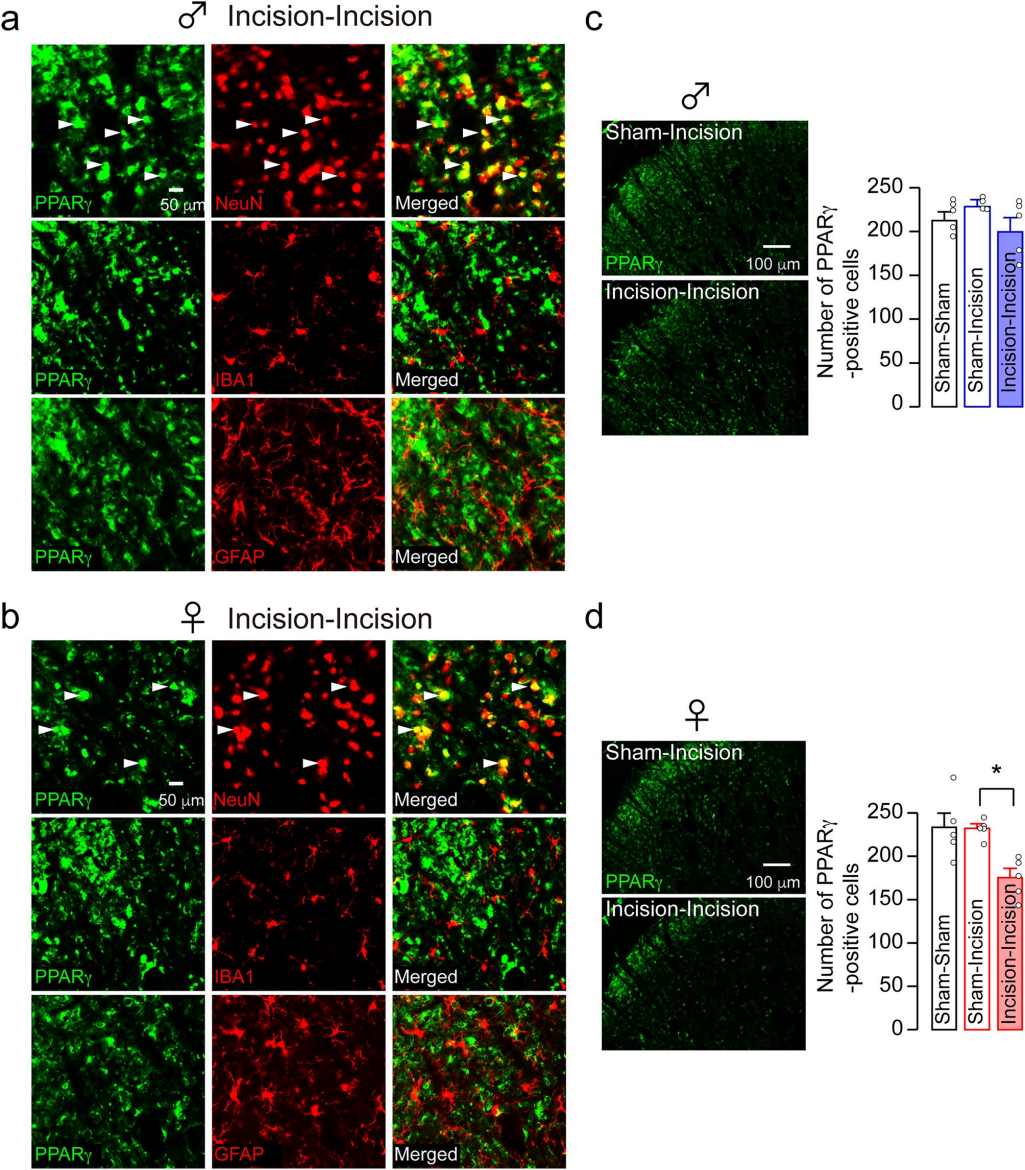
Expression patterns of PPARγ in the Vc. (**a, b**) The images showing PPARγ, NeuN, IBA1, and GFAP immunofluorescence in the Vc of the Incision-Incision group in male rats (**a**) and female rats (**b**) 10 days after whisker pad skin re-incision. Arrowheads indicate PPARγ and NeuN double-positive cells. Scale bars = 50 µm. (**c, d**) The representative images of PPARγ immunofluorescence in the Vc of male (**c**) and female rats (**d**) 10 days after whisker pad skin re-incision. The columns represent the mean number of PPARγ-positive cells in the Vc 10 days after re-incision. Scale bars = 100 µm. Data represent the mean ± SEM. n = 5 in each, one-way ANOVA post hoc Tukey’s test, *p = 0.0105 in (**d**).

**Figure 5:**
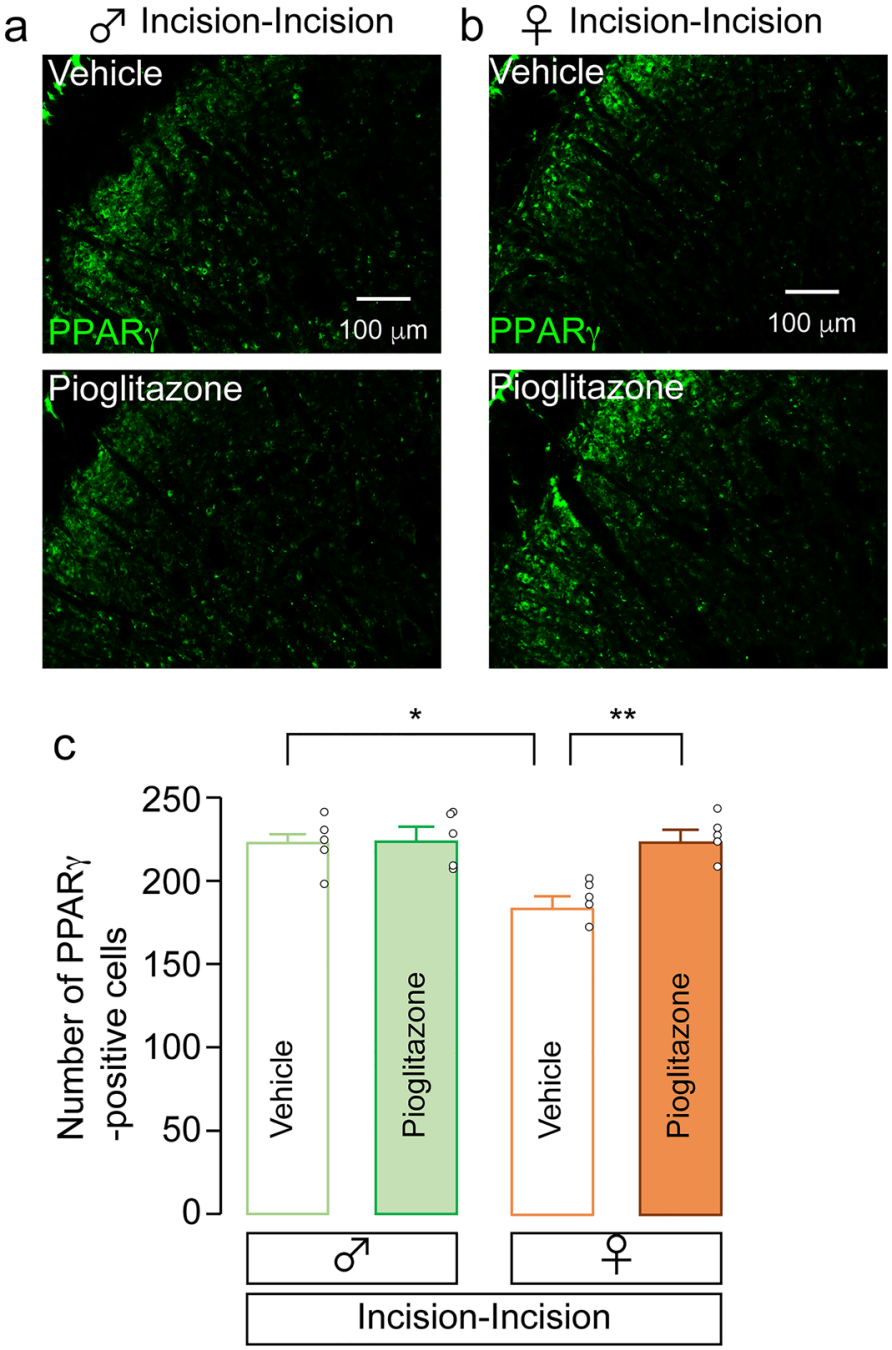
The effects of pioglitazone on the number of PPARγ-positive cells in the Vc of Incision-Incision rats. (**a, b**) The representative images of PPARγ immunofluorescence in the Vc of male (**a**) and female rats (**b**) 10 days after whisker pad skin re-incision. Scale bars = 100 µm. (**c**) The columns represent the mean number of PPARγ-positive cells in the Vc 10 days after whisker pad skin re-incision. Data represent the mean ± SEM. n = 5 in each, two-way ANOVA post hoc Tukey’s test, *p = 0.0105, **p = 0.0030.

We next evaluate the distribution of PPARγ in the Vc using immunohistochemical analyses. At 10 days after skin re-incision, PPARγ immunofluorescence in the Vc of both sexes was exclusively merged with NeuN, a marker of neuron, but not with glial fibrillary acidic protein (GFAP, a marker of astrocyte) and IBA1 (Fig. 4a,b), suggesting that PPARγ localized in Vc neurons. The number of PPARγ-positive Vc neurons of male rats was unaltered by skin re-incision (one-way ANOVA post hoc Tukey’s test, *p* = 0.1381, F_(2,12)_ = 1.635; Fig. 4c). However, that of female rats was significantly reduced by skin re-incision (one-way ANOVA post hoc Tukey’s test, *p* = 0.0048, F_(2,12)_ = 1.352; Fig. 4d). We further evaluated the effects of pioglitazone, an agonist for PPARγ, on the number of PPARγ-positive Vc neurons. In male rats, pioglitazone had no effects on the number of PPARγ-positive Vc neurons in the Incision-Incision group (two-way ANOVA post hoc Tukey’s test, *p* = 0.9921; Fig. 5a,c). However, in female rats, that was significantly increased by pioglitazone administration in the Incision-Incision group (two-way ANOVA post hoc Tukey’s test, *p* = 0.0030; Fig. 5b,c). In the vehicle-treated group, the number of PPARγ-positive Vc neurons in female rats was significantly lower than that in male rats (two-way ANOVA post hoc Tukey’s test, *p* = 0.0105; Fig. 5c).

### Effect of pioglitazone on antioxidant enzymes

**Figure 6:**
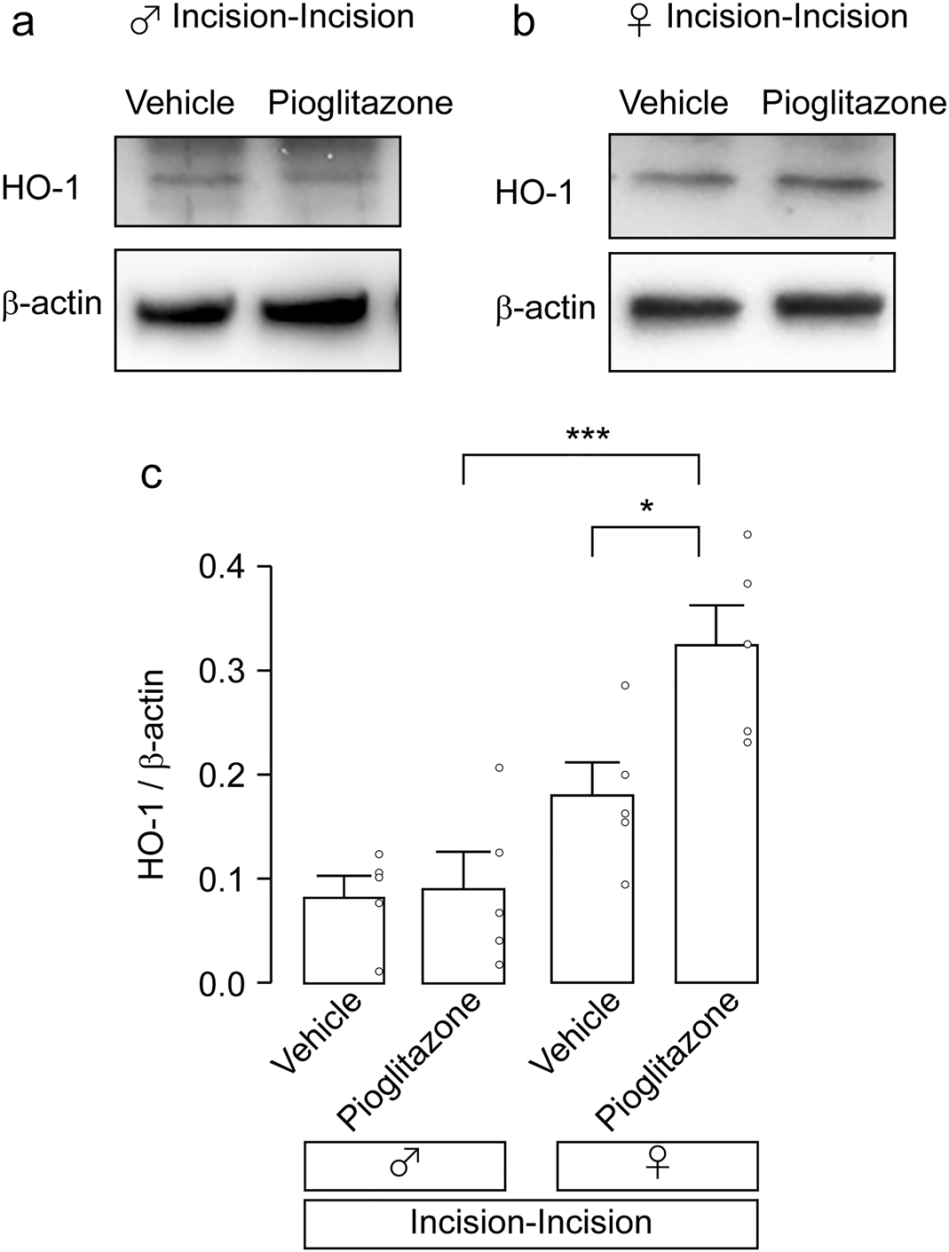
The effects of pioglitazone on HO-1 expression in the Vc of incision-incision rats. (**a, b**) Representative blot of HO-1 expression in the Vc of male (**a**) and female rats (**b**) 10 days after whisker pad skin re-incision. Original blots are presented in Supplementary Figs. 1 and 2. (**c**) The columns represent the average value of HO-1/β-actin. Data represent the mean ± SEM. n = 5 in each, two-way ANOVA post hoc Tukey’s test *p = 0.0271, ***p = 0.006.

A representative role of PPARγ is the modulation of redox activity^17^. We thus expected that pioglitazone might affect the amount of reactive oxygen species (ROS) in the Vc in the Incision-Incision group. We focused on antioxidant enzymes that might be regulated by pioglitazone. Heme oxygenase 1 (HO-1) is a stress-inducible molecule that suppresses ROS production^18^. The amount of HO-1 in the Vc in the Incision-Incision group was not influenced by intracisternal administration of pioglitazone in male rats (two-way ANOVA post hoc Tukey’s test, *p* = 0.9975; Fig. 6a,c). On the other hand, in female rats, that was increased by intracisternal administration of pioglitazone (two-way ANOVA post hoc Tukey’s test, *p* = 0.0271; Fig. 6b,c). HO-1 expression in pioglitazone-treated female rats was significantly higher than that in male rats (two-way ANOVA post hoc Tukey’s test, *p* = 0.006, Fig. 6c). We further analyzed the enzymatic activity of superoxide dismutase (SOD). However, no significant change in the SOD activity was observed after pioglitazone administration in the Incision-Incision group (male vehicle: 64.73 ± 2.24%; male pioglitazone: 59.11 ± 4.35%, unpaired t-test, *p* = 0.2840, t_(8)_ = 1.148; female vehicle: 46.92 ± 7.40%; female pioglitazone: 45.01 ± 5.49%, unpaired t-test, *p* = 0.8411, t_(8)_ = 0.2072).

### Effect of pioglitazone on the expression levels of N-methyl-_D_-aspartate receptors (NMDARs)

**Figure 7:**
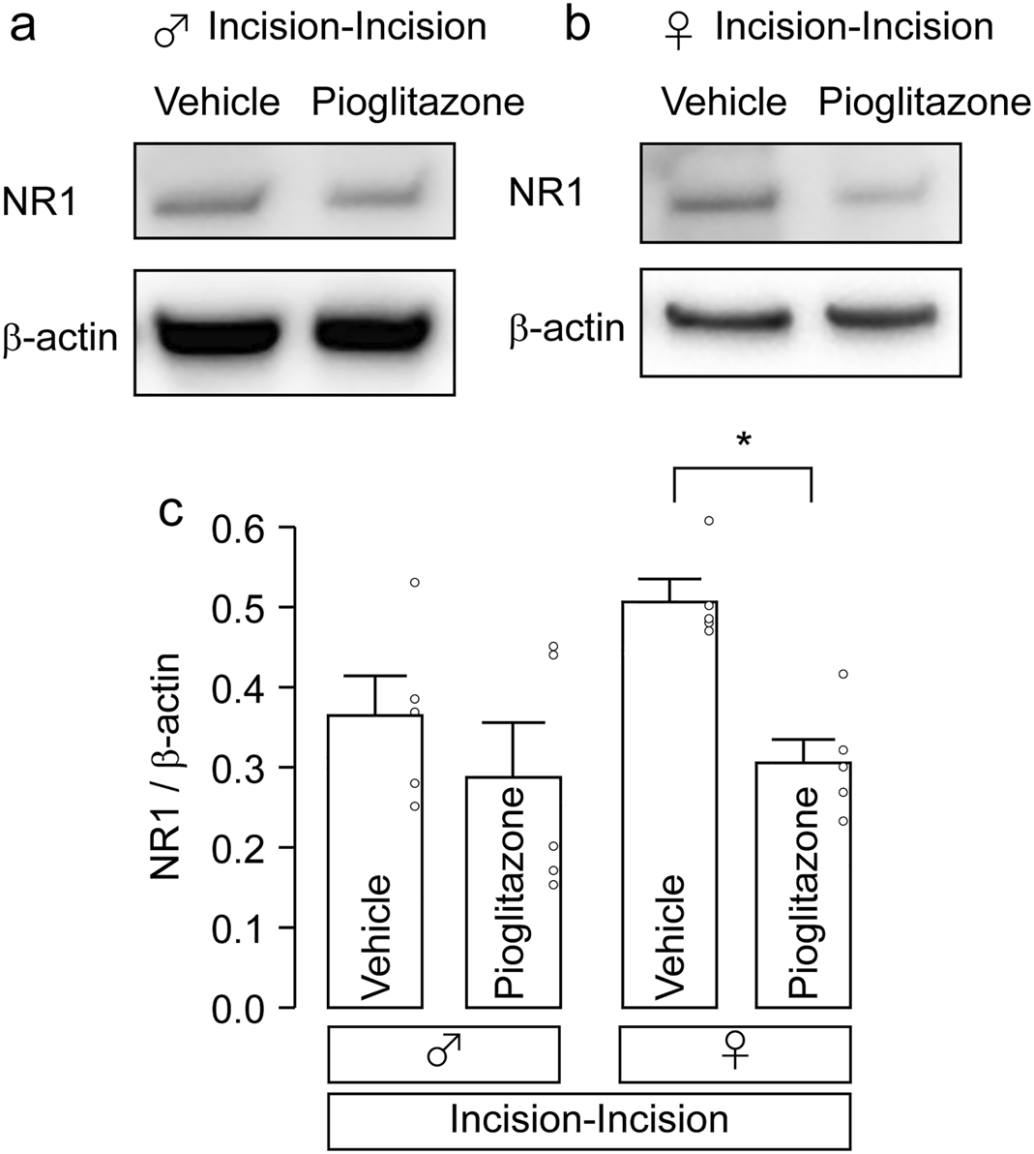
The effects of pioglitazone on NR1 expression in the Vc of incision-incision rats. (**a, b**) Representative blot of NR1 expression in the Vc of male (**a**) and female rats (**b**) 10 days after whisker pad skin re-incision. Original blots are presented in Supplementary Figs. 3 and 4. (**c**) The columns represent the average value of NR1/β-actin. Data represent the mean ± SEM. n = 5 in each, two-way ANOVA post hoc Tukey’s test, *p = 0.0269.

NMDARs are hetero-oligomeric proteins composed of an obligatory NR1 subunit and one or two kinds of NR2 subunits^19^. NR1 is known as the essential subunit of the NMDARs and it will have a significant impact on the functionality of all NMDAR variants^20^. Accordingly, the amount of NR1 in the Vc was analyzed. In male rats, the expression levels of NR1 in the Vc were unchanged by intracisternal administration of pioglitazone in the Incision-Incision group (two-way ANOVA post hoc Tukey’s test, *p* = 0.6272; Fig. 7a,c). In contrast, those in female rats were significantly reduced by intracisternal administration of pioglitazone (two-way ANOVA post hoc Tukey’s test, *p* = 0.0269; Fig. 7b,c).

## Discussion

In the present study, we found that incision-induced mechanical allodynia in the whisker pad skin was prolonged by neonatal incision in both male and female rats. In female rats, microglia are not implicated in the prolongation of the incision-induced mechanical allodynia due to neonatal incision. An increased number of T cells was observed in the Vc after re-incision in female rats; however, it was not dramatic. Intracisternal administration of pioglitazone markedly suppressed the mechanical allodynia after whisker pad skin re-incision in female rats. However, the suppressing effect of pioglitazone was weak in male rats. Expression of PPARγ in the Vc was restricted in neurons. Following whisker pad skin re-incision, the number of PPARγ-positive Vc neurons was significantly reduced in female rats but not in male rats. PPARγ-positive Vc neurons in female rats after whisker pad skin re-incision was increased by intracisternal administration of pioglitazone. The expression levels of HO-1 and NR1 subunit in the Vc were upregulated and downregulated, respectively, by intracisternal administration of pioglitazone in the Incision-Incision group in female rats. These results suggest that the prolonged mechanical allodynia due to whisker pad skin re-incision in female rats is attributed to a decrease in PPARγ in Vc neurons, a concomitant decrease in HO-1, and an increase in NR1 subunit.

Sex dimorphism in the morphological changes of Vc microglia were observed after re-incision. In male rats, soma size and cell density of Vc microglia were increased after re-incision, whereas arborization area was unchanged. Similarly, soma size but not arborization area of Vc microglia was increased in female rats. The mechanisms underlying the sex difference in morphological changes of microglia after re-incision remain unclear. Recent transcriptomic analyses from male and female microglia clarified distinct gene profiles. Male microglia have higher levels of transcription of genes associated with inflammatory processes such as cytokine production and cell migration than female microglia^21,22^. Indeed, in the ischemic model, male microglia facilitate neuroinflammation, whereas female microglia protect it^21^. In pain models, inhibition of microglia-specific molecules such as P2X4R, p38 mitogen-activated protein kinase, and BDNF had an effect in male mice but not in female mice^8,23,24^. In addition, mechanical allodynia was unaltered by microglial ablation in female mice^8^. Conversely, selective chemogenic activation of microglia with clozapine N-oxide elicits mechanical allodynia in male CX3CR1-hM3Dq mice but not in female mice^15^. Furthermore, upregulation of proinflammatory cytokines was observed only in male mice^15^. Given this information, it is possible that microglia in male and female animals have distinct phenotypes and may differ in responsiveness. Vc microglia in female rats might not be an inflammatory phenotype after re-incision. Minocycline is widely used in glial research to inhibit the transformation of microglia toward an inflammatory phenotype^8,16^ and does not affect microglial ramification or IBA1 expression under normal conditions^25,26^. Therefore, it is possible that minocycline unaffected Vc microglia in female rats even after re-incision.

Accumulating evidence indicates that inflammatory factors from microglia are the essential factor to develop neuropathic pain in male animals but not in female animals^8,15,16^. In contrast, infiltrated T cells in the spinal dorsal horn are reported to contribute to neuropathic pain in female animals^8,16^. In the Vc, we found the prolonged incision-induced mechanical allodynia in male rats was dependent on microglia, consistent with the previous report^4^. Although CD3-positive cells were seen in the Vc after whisker pad skin re-incision in both sexes, the number of CD3-positive cells was very small, suggesting that infiltrated T cells in the Vc after whisker pad skin re-incision are unlikely to be involved in prolonged incision-induced mechanical allodynia in female rats. Indeed, there were only a few GFP and CD3 double-positive cells in the spinal cord of bone marrow chimeric mice transplanted with bone marrow from CAG-EGFP mice^12^. However, due to the technical limitation of T cell ablation in rats, we can not rule out the possible involvement of infiltrated T cells in the Vc in the prolongation of incision-induced mechanical allodynia in female rats.

It has been reported that the adoptive transfer of T cells from mice with neuropathic pain elicited mechanical allodynia in naïve mice^9,27^, indicating the possible involvement of circulating activated T cells in neuropathic pain. Recent findings revealed the involvement of accumulated immune cells such as T cells and macrophages in the dorsal root ganglion (DRG) exacerbated neuropathic pain^11,28^. Considering the lack of blood-brain barrier in the trigeminal ganglion (TG)^29^, circulating T cells might be accumulated in the TG following skin re-incision in female rats and then potentiate neuronal activity in the TG. Nevertheless, the effect of pioglitazone on the TG is very unlikely given that we administrated it in the cisterna magna but not in the TG. In the future, we should also investigate the involvement of T cells in the TG on incision-induced mechanical allodynia in female rats. Currently, information on circulating T cells in chronic pain patients is limited and has only been studied in the blood of chronic headache and complex regional pain syndrome^30,31^. Future clinical studies will reveal more details about circulating T cells during chronic pain.

Following whisker pad skin re-incision, PPARγ expression in the Vc was decreased in female rats with neonatal incision compared with male rats, which might be attributed to sex hormones, given that estrogen facilitates PPARγ expression^32^. Another PPARγ property is known that PPARγ expression is enhanced by its own stimulation^33^. In the clinical study, it has been suggested that psychological stress may reduce blood estrogen levels^34^. Based on these facts, it is presumed that the stress caused by neonatal tissue injury may have primed and reduced blood estrogen levels in response to secondary injury in adulthood, leading to a reduction in the number of PPARγ-positive Vc neurons in female rats. Postmenopausal changes in pain sensitivity^35^ may be due to decreased expression of PPARγ in secondary neurons associated with decreased estrogen levels. PPARγ agonist efficiently ameliorates mechanical allodynia caused by spared nerve injury or spinal cord injury in female animals^8,14^. Although evidence for PPARγ expression levels in secondary neurons has not yet been reported in these models, they would likely be upregulated by PPARγ agonists. Thus, a decrease in PPARγ expression in the Vc after skin re-incision causes the prolongation of incision-induced mechanical allodynia in female rats.

PPARγ is involved in a wide variety of functions including adipogenesis, glucose metabolism, and bone morphogenesis as a transcription factor^13^. In particular, PPARγ facilitates the transcription of antioxidant enzymes such as HO-1, manganese SOD, and catalase^17^. In the present study, we found that HO-1 expression in the Vc was increased by intracisternal administration of pioglitazone in the Incision-Incision group in female rats. However, SOD activity was unchanged by pioglitazone administration. Though details as to why SOD activity was unaffected by pioglitazone administration are currently unknown, these data suggest that excessive ROS production leads to prolonged incision-induced mechanical allodynia in the Incision-Incision group in female rats.

Given the above evidence, ROS might contribute to the prolongation of incision-induced mechanical allodynia in female rats. In our experiment, ROS produced in Vc neurons might affect neuronal activity. The possible involvement of ROS in pain has been reported in several previous studies^36,37^. Following the L5 nerve transection, microglia produce ROS which increases the production of pro-inflammatory cytokines in microglia, leading to neuropathic pain^37^. However, the fact that microglia contribute less to neuropathic pain in female animals suggests that ROS has less effect on microglia in female rats after skin re-incision. In terms of the effect of ROS on neurons, phosphorylation of the NR1 subunit has been reported^36,38^. Distinct from these data, we detected the reduction of NR1 subunit in the Vc after pioglitazone administration in the Incision-Incision group in female rats. Whereas, the phosphorylated NR1 subunit was unchanged by pioglitazone administration (data not shown). Transcription of NR1 is regulated by nuclear factor-kappa B (NF-κB) that binds to the promoter site of NR1 subunit^39^. Besides, ROS can activate NF-κB signaling^40^. Accordingly, pioglitazone may reduce ROS production and subsequent transcription of NR1 subunit in the Vc of the Incision-Incision group in female rats.

NMDA receptors are composed of an obligatory NR1 subunit and four kinds of NR2 subunits. Excessive activation of NR1 or NR2B subunits exacerbates mechanical allodynia in the orofacial region^41,42^. NR1 subunit is known as the glycine binding site, which modulates ion influx through NMDA receptors^20,43^. Activation of NMDA receptors causes Ca^2+^ influx which leads to the formation of long-term potentiation in lamina I spinal neurons^44^. NR1 in the Vc might be upregulated after whisker pad skin re-incision. Therefore, pioglitazone presumably suppresses the formation of long-term potentiation in Vc neurons of the Incision-Incision group in female rats. Other targets of pioglitazone might be GABARs. It is reported that pioglitazone upregulates the expression of GABARa2 in hippocampal CA1 neurons^45^, leading to the facilitation of inhibitory synaptic transmission. ROS which is a target of pioglitazone reduces the GABAergic input in substantia gelatinosa neurons in the spinal cord^38^. Thus, it is possible that pioglitazone also enhances the inhibitory neurotransmission, thereby suppressing incision-induced mechanical allodynia in female rats.

Taken together, pioglitazone suppressed pain prolongation by increasing the number of PPARγ-positive Vc neurons and the expression of HO-1, and by reducing the expression of NR1 subunit in female rats. This experiment allowed us to identify a pain mechanism unique to female rats that differs from that of male rats. Based on the current findings, we propose the need for different analgesic treatments according to gender.

## Experimental procedures

### Animals

Pregnant Sprague-Dawley rats (n = 14) were purchased from Japan SLC (Hamamatsu, Japan) and rats were used for experiments after birth. A total of 45 male littermates and 45 female littermates were used for the experiments. Rats were maintained at 23 ± 1 °C in 12 h light/dark cycle (light on 7:00–19:00) with food and water ad libitum. The animal experiment was approved by the experimentation committee at Nihon University (protocol number: AP20DEN013). All animal care was in compliance with the guidelines of the International Association for the Study of Pain^46^. Animal studies are reported in compliance with the guidelines of the Animal Research: Reporting of In Vivo Experiments.

### An incision in the whisker pad skin

Skin incision (2.5 mm length, 1 mm depth) was made in the left whisker pad of the rat on neonatal (postnatal day 4) under inhalation anesthesia with 2% isoflurane (Mylan, Canonsburg, PA, USA). Then, the incision was sutured with 6–0 silk thread (Natsume, Tokyo, Japan). For the sham operation, the left whisker pad skin was sutured without incision. At 7 weeks of age, rats with neonatal incision suffered skin incision (10 mm length, 1 mm depth) in the same region of the whisker pad under inhalation anesthesia with 2% isoflurane. Skin incision was performed using a number 11 blade scalpel.

### Cannula implantation and drug administration

Two days before an incision in adulthood, a hole (approximately 1 mm diameter) was made in the occipital bone and the tip of polyethylene tubing (SP10; Natsume) was placed in the cisterna magna under deep anesthesia with a mixture of butorphanol (2.5 mg/kg; Meiji Seika Pharma, Tokyo, Japan), midazolam (2.0 mg/kg; Sandoz, Tokyo, Japan), and medetomidine (0.15 mg/kg; Zenoaq, Koriyama, Japan), intraperitoneally. The following day, an osmotic pump (model 1002; Alzet, Cupertino, CA, USA) filled with saline, minocycline (1 fmol /h, Sigma-Aldrich, St Louis, MO, USA), or pioglitazone (8 fmol /h, FUJIFILM Wako Pure Chemicals Corporation, Osaka, Japan) was connected to the cannula.

### Measurement of head withdrawal threshold

All rats were acclimated to the testing environment for 5 days. The von Frey filaments (4, 6, 8, 10, 15, 26, 30, 40, 50, 60, and 80 g) were applied to the left whisker pad skin according to the method described previously^47^. Each filament was used ascending manner. When the rats exhibited head withdrawal response by von Frey stimulation at least 3 out of 5 times, we defined that rats exhibited nociceptive response. The lowest intensity stimulus that produced a nociceptive response was determined to be HWT.

### Immunohistochemistry

The rats were deeply anesthetized with 5% isoflurane inhalation after the termination of behavioral testing. Then, the rats were transcardially perfused with ice-cold saline, followed by 4% paraformaldehyde (PFA) in 0.1 M phosphate buffer. The brainstem segment was excised, further fixed in 4% PFA overnight at 4 °C, and placed in 30% sucrose for 48 h at 4 °C. Forty-micrometer-thick sections of the Vc were made according to the method described previously^41^. The slices were blocked by 0.1 M phosphate-buffered saline (PBS) containing 0.4% Triton X-100 (FUJIFILM Wako Pure Chemicals Corporation), 1% donkey serum (Jackson ImmunoResearch, West Grove, PA, USA), and 1% bovine serum albumin (Proliant Biologicals, Boone, IA, USA) for 1 h at room temperature. The slices were incubated 2 days at 4 °C with primary antibodies for IBA1 (rabbit polyclonal, 1:5000, Cat.No. 019-19741; FUJIFILM Wako Pure Chemicals Corporation), PPARγ (rabbit polyclonal, 1:50, Cat.No. 16643-1-AP; Proteintech, Rosemont, IL, USA), or CD3 (mouse monoclonal, 1:200, Cat.No. MCA772; Bio-Rad, Hercules, CA, USA), GFAP (mouse monoclonal, 1:2000, Cat.No. MAB360; Merck Millipore), or NeuN (mouse monoclonal, 1:5000; Cat.No. MAB377; Merck Millipore, Billerica, MA, USA). After gentle washing, the slices were incubated with secondary antibodies conjugated with Alexa Fluor 488 or Alexa Fluor 568 (1:400 in each; Thermo Fisher Scientific, Cleveland, OH, USA) for 2 h at 4 °C. The slices were mounted in PermaFluor Aqueous Mounting Medium (Thermo Fisher Scientific). Images were captured by a BZ-9000 (Keyence, Osaka, Japan). Signals that are at least twice as strong as the background signal are considered the signal of positive. The region of interest (466 × 620 µm^2^) was set in the Vc region where the 2nd branch was terminated. The number of IBA1-positive cells or PPARγ-positive cells were counted using Image J software (NIH; http://rsbweb.nih.gov/ij/). Three slices from each animal were stained, the number of IBA1-positive cells or PPARγ-positive cells was counted, and the average of the counts was used. The density of IBA1-positive cells in the Vc was calculated as cell density per area (100 × 100 µm^2^).

### Morphological analyses of microglia

The soma area and ramification were assessed from the images according to the method described previously^48^. The soma area was measured by drawing a line around the soma with a freehand selection tool of ImageJ software. The ramification index of microglia was measured by the area of the polygon obtained by connecting the tips of each process with a line was measured. Twenty microglia from one slice were analyzed, and three slices were measured and averaged for each rat.

### Western blotting

After the cessation of behavioral testing, the rats were perfused with ice-cold saline under 5% isoflurane anesthesia. The Vc section was quickly excised and the Vc region where the 2nd branch of the trigeminal nerve is terminated was excised. Sections were stored at − 80 °C until use. The sample was lysed in lysis buffer (10 mM Tris-HCl, pH 7.4, 150 mM NaCl, 1% Triton X-100, and 0.5% NP-40) supplemented with 1% protease inhibitor cocktail (Sigma-Aldrich) and 1% phosphatase inhibitor (Nacalai Tesque, Kyoto, Japan). Protein concentration was measured by a bicinchoninic acid assay kit (Takara, Otsu, Japan). Proteins (5–10 µg) were mixed with 2 × Laemmli buffer supplemented with 2-mercaptoethanol, and denatured for 5 min at 95 °C. Samples were loaded in a 4–20% Mini-PROTEAN TGX Precast Gel (Bio-Rad). After electrophoresis, proteins were transferred onto polyvinylidene difluoride membrane (Trans-Blot Turbo Transfer Pack, Bio-Rad). Blocking was performed using 5% Blocking One-P in TBS-T (0.2% Tween-20 diluted in Tris-buffered saline) for 1 h at room temperature. The membrane was incubated overnight at 4 °C with primary antibodies for anti-NR1 antibody (mouse monoclonal, 1:800, Cat.No.05–432; Merck Millipore), anti-HO-1 antibody (rabbit polyclonal, 1:1000, Cat.No.70081S; Cell Signaling Technology, Beverly, MA, USA), and anti-β-actin antibody (mouse monoclonal, 1:200; Cat.No. sc-69879; Santa Cruz San Diego, CA, USA). After a gentle wash with TBS-T, the blots were incubated with horseradish peroxidase (HRP)-conjugated secondary antibodies for anti-mouse IgG or anti-rabbit IgG (1:2000, Cat.No.NA931V or Cat.No. NA934V; Cytiva, Marlborough, MA, USA) 2 h at room temperature. HRP was detected by the Western Lightning ELC Pro (PerkinElmer, Waltham, MA, USA). The images were captured by a ChemiDocXRS system (Bio-Rad). The band intensity was quantified using ImageJ and normalized to β-actin.

### SOD assay

The Vc section was lysed in lysis buffer with the same composition as above. SOD activity in Vc lysate (proteins: 5 µg) was assessed using SOD assay kit (Dojindo, Tokyo, Japan) according to the manufacturer’s protocol.

### Statistical analyses

Reagent selection, behavioral testing, immunohistochemistry, Western blotting, and statistical analyses were separately and blindly conducted. Data are presented as the median ± interquartile range or the mean ± standard error of mean (SEM), as appropriate. Data normality was assessed using the Shapiro–Wilk test. The statistical analyses were performed using an unpaired Student’s t-test or one-way analysis of variance (ANOVA) post hoc Tukey’s test using the GraphPad Prism 9 software program (GraphPad Software Inc., San Diego, CA, USA). Data from the behavioral experiments were subjected to nonparametric tests, and generalized estimated equation post hoc Bonferroni’s test or Freidman post hoc Dunn’s test were used to perform repeated testing with SPSS version 28 (IBM, New York, NY, USA). Differences were considered to be significant for values at *P* < 0.05.

## Ethical approval

Animal experiment was approved by the Experimentation Committee of Nihon University (Protocol number: AP20DEN013).

## Supplementary Information

Supplementary Information.

## Data Availability

The data used in this study are available from the corresponding authors upon reasonable request.
