# Supplementary material for: Decreased PPARgamma in the trigeminal spinal subnucleus caudalis due to neonatal injury contributes to incision-induced mechanical allodynia in female rats
Source: Sci Rep. 2022 Nov 11;12:19314. doi: 10.1038/s41598-022-23832-3 (PMC9652333; doi:10.1038/s41598-022-23832-3)
Supplement: Supplementary file 1 — Supplementary Information. [file 41598_2022_23832_MOESM1_ESM.pdf]

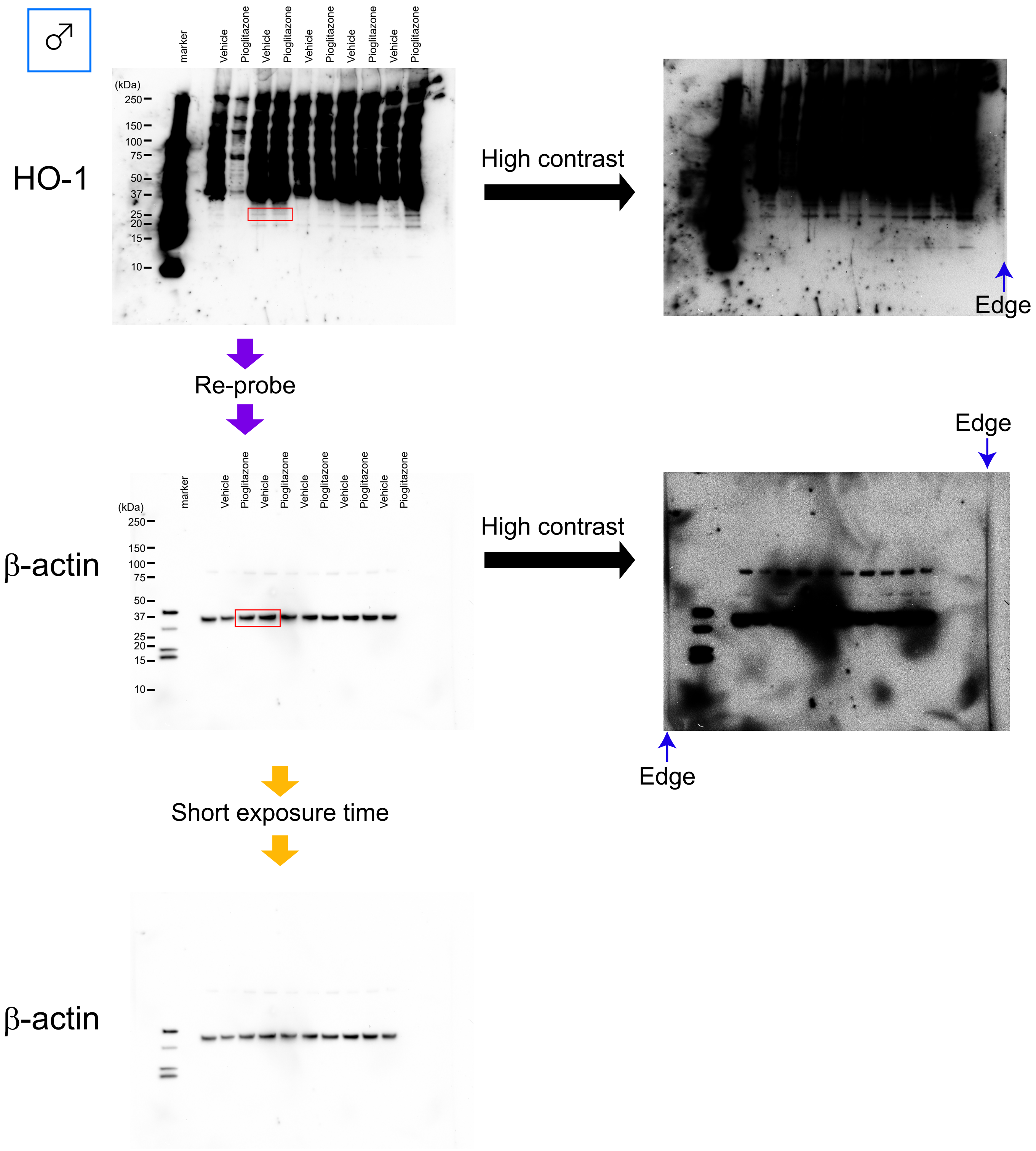

Supplementary Figure 1.

Left showing full-length images of blots in Figure 6a.

All replicates are included in the blot. Right images showing high-contrast images of the original images to visible the membrane edges. Blue arrows indicate the membrane edges. The lower left image is β-actin image taken with a short exposure time. The area enclosed by the red line is used in the figures.

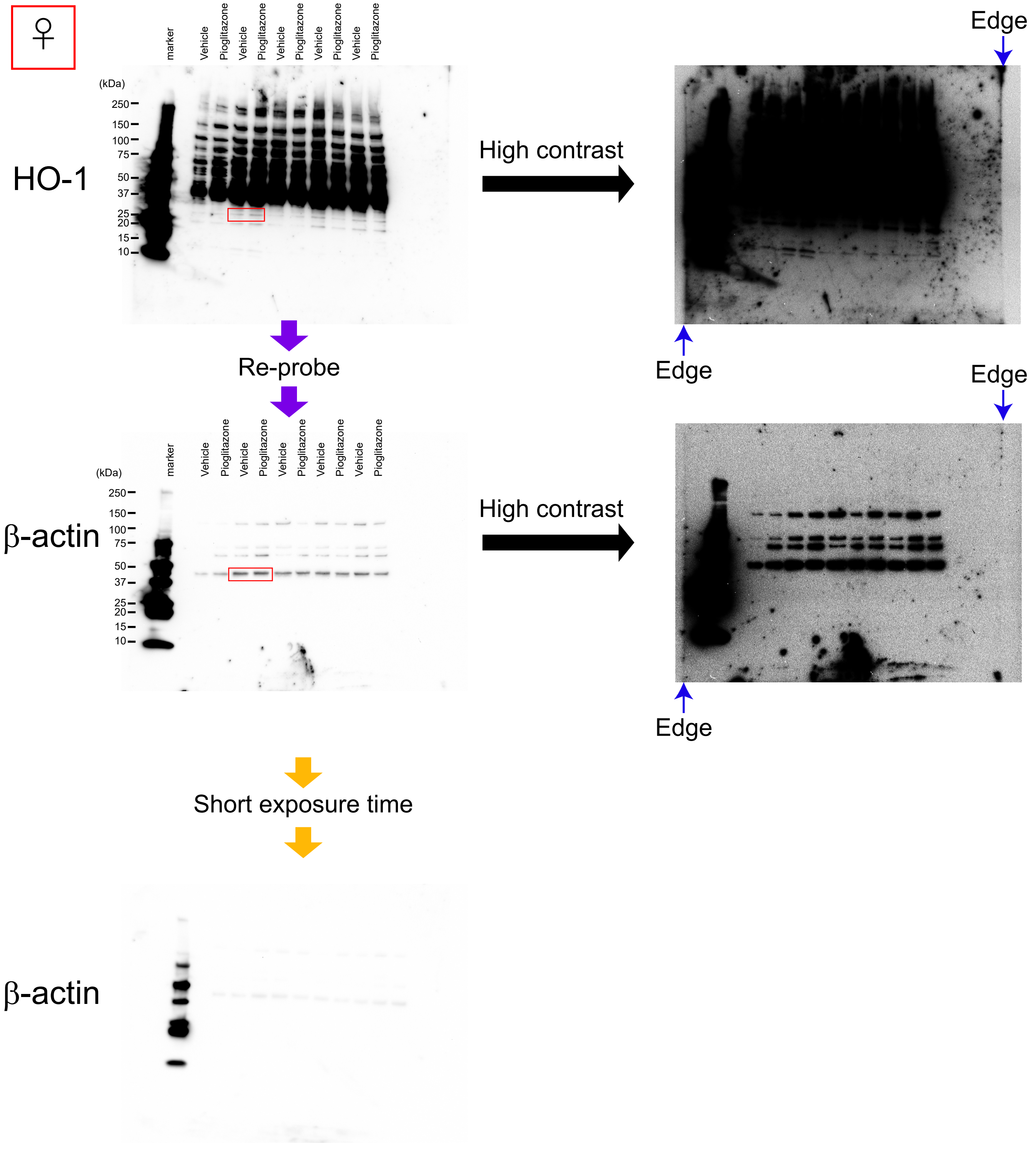

Supplementary Figure 2.

Left showing full-length images of blots in Figure 6b.

All replicates are included in the blot. Right images showing high-contrast images of the original images to visible the membrane edges. Blue arrows indicate the membrane edges. The lower left image is β-actin image taken with a short exposure time. The area enclosed by the red line is used in the figures.

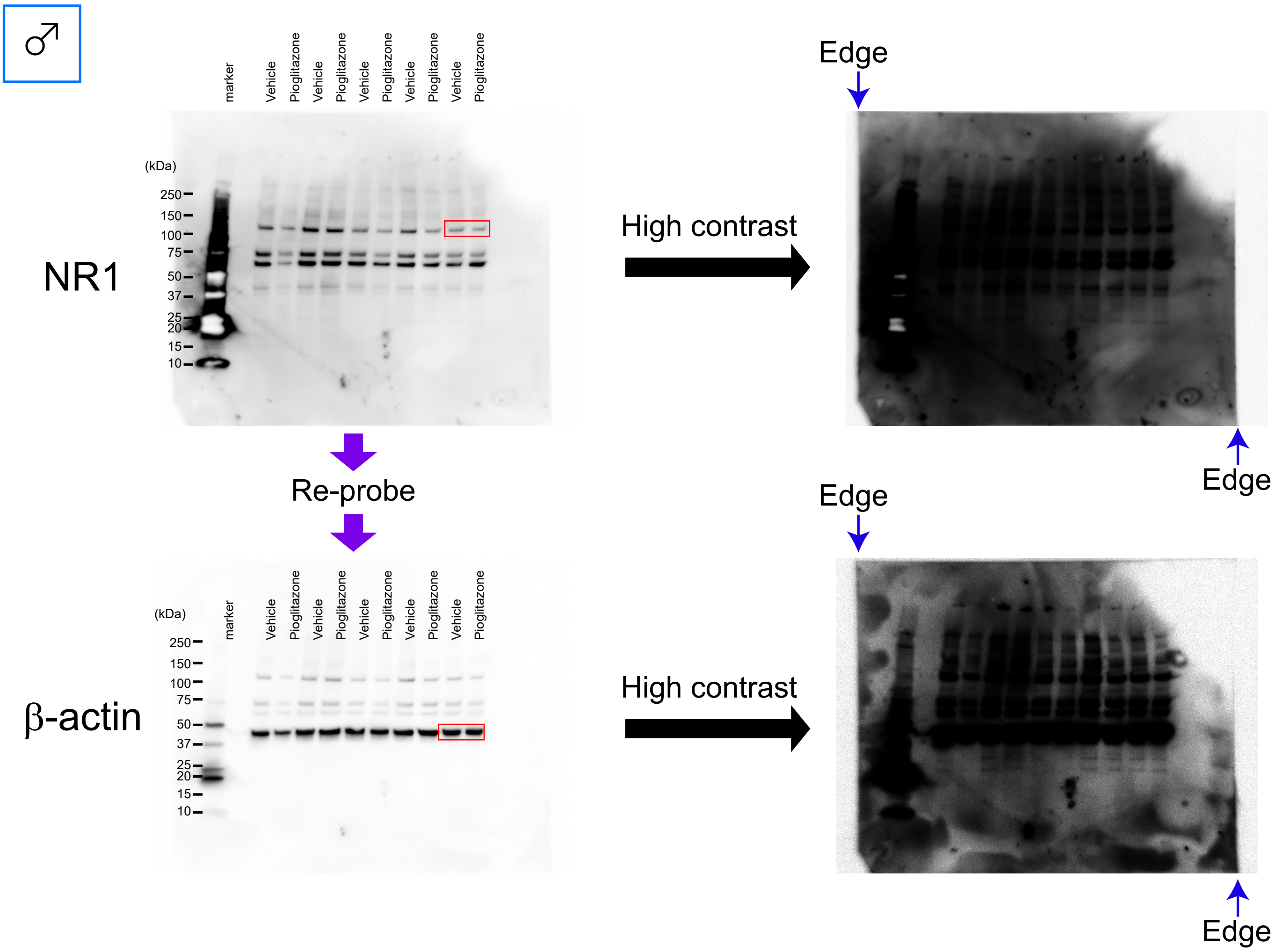

Supplementary Figure 3.

Left showing full-length images of blots in Figure 7a.

All replicates are included in the blot. Right images showing high-contrast images of the original images to visible the membrane edges. Blue arrows indicate the membrane edges. The area enclosed by the red line is used in the figures.

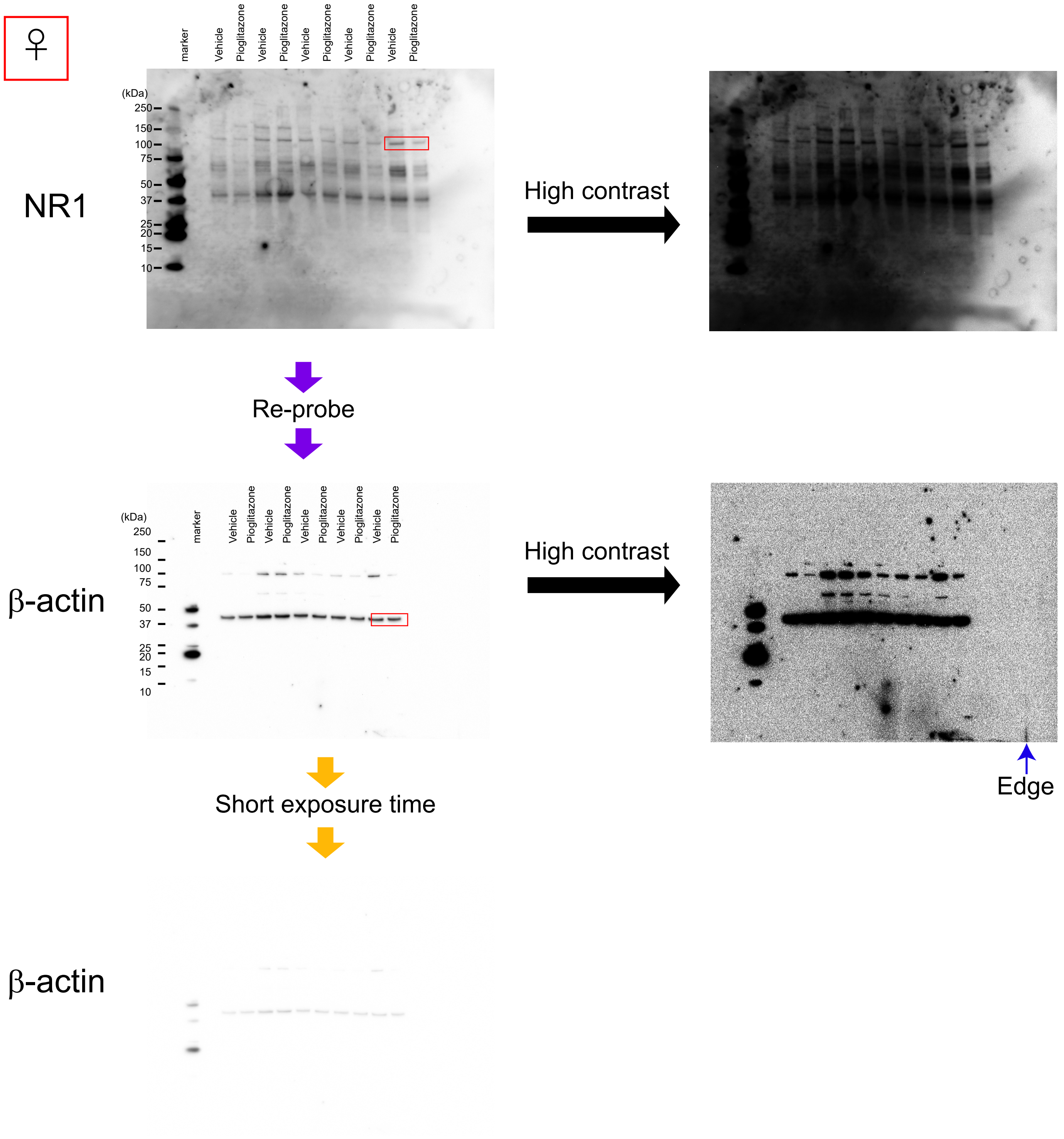

Supplementary Figure 4.

Left showing full-length images of blots in Figure 7b.

All replicates are included in the blot. Right images showing high-contrast images of the original images to visible the membrane edges. Blue arrows indicate the membrane edges. The lower left image is β-actin image taken with a short exposure time. The area enclosed by the red line is used in the figures.
